# Effects of Different No-Ozone Cold Plasma Treatment Methods on Mouse Osteoblast Proliferation and Differentiation

**DOI:** 10.3390/medicina60081318

**Published:** 2024-08-14

**Authors:** Byul-Bo Ra Choi, Sang-Rye Park, Gyoo-Cheon Kim

**Affiliations:** 1 Corporate Affiliated Research Institute, Feagle Co., Ltd., Yangsan 50561, Republic of Korea; cbbrstar@feagle.co.kr; 2 Department of Dental Hygiene, Kyungnam College of Information & Technology, Busan 47011, Republic of Korea; sanglye5@naver.com; 3 Department of Oral Anatomy, School of Dentistry, Pusan National University, Yangsan 50612, Republic of Korea

**Keywords:** alkaline phosphatase, mouse osteoblast, no-ozone cold plasma, osteoblast differentiation, osteocalcin

## Abstract

*Background and Objectives*: Enhanced osteoblast differentiation may be leveraged to prevent and treat bone-related diseases such as osteoporosis. No-ozone cold plasma (NCP) treatment is a promising and safe strategy to enhance osteoblast differentiation. Therefore, this study aimed to determine the effectiveness of direct and indirect NCP treatment methods on osteoblast differentiation. Mouse osteoblastic cells (MC3T3-E1) were treated with NCP using different methods, i.e., no NCP treatment (NT group; control), direct NCP treatment (DT group), direct NCP treatment followed by media replacement (MC group), and indirect treatment with NCP-treated media only (PAM group). *Materials and Methods*: The MC3T3-E1 cells were subsequently assessed for cell proliferation, alkaline phosphatase (ALP) activity, calcium deposition, and ALP and osteocalcin mRNA expression using real-time polymerase chain reaction. *Results*: Cell proliferation significantly increased in the NCP-treated groups (DT and PAM; MC and PAM) compared to the NT group after 24 h (*p* < 0.038) and 48 h (*p* < 0.000). ALP activity was increased in the DT and PAM groups at 1 week (*p* < 0.115) and in the DT, MC, and PAM groups at 2 weeks (*p* < 0.000) compared to the NT group. Calcium deposition was higher in the NCP-treated groups than in NT group at 2 and 3 weeks (*p* < 0.000). ALP mRNA expression peaked in the MC group at 2 weeks compared to the NP group (*p* < 0.014). Osteocalcin mRNA expression increased in the MC group at 2 weeks (*p* < 0.000) and was the highest in the PAM group at 3 weeks (*p* < 0.000). Thus, the effects of direct (DT and MC) and indirect (PAM) treatment varied, with MC direct treatment showing the most significant impact on osteoblast activity. *Conclusions*: The MC group exhibited enhanced osteoblast differentiation, indicating that direct NCP treatment followed by media replacement is the most effective method for promoting bone formation.

## 1. Introduction

Osteoporosis is a systemic skeletal disease that increases the risk of bone fractures, owing to decreased bone density and mass [1]. According to the International Osteoporosis Foundation, one in five women and one in five men older than 50 years worldwide have osteoporosis [2]. The main causes of osteoporosis are aging and, in women, a rapid decrease in estrogen levels owing to menopause [3]. Therefore, the prevention and treatment of osteoporosis are crucial.

Bone tissue comprises hydroxyapatite crystals, collagen, and non-collagenous proteins [4]. Most bone-related diseases are associated with decreased expression of factors related to bone formation [5]. Additionally, hormonal imbalances during bone remodeling can lead to bone loss [6] Consequently, maintaining bone homeostasis, which requires a balance between osteoblasts (bone-forming cells) and osteoclasts (bone-absorbing cells), is crucial for preventing osteoporosis [7]. Current treatments for osteoporosis include drug therapy; however, they have a limited effect in terms of long-term safety [8,9]. In contrast, recent studies suggest that enhancing osteoblast differentiation may represent a new method for preventing and treating osteoporosis [10].

Cold plasma, the fourth state of matter after solids, liquids, and gases, is being extensively studied for its medical applications [11]. Studies have investigated its use in killing bacteria [12], regenerating damaged nerves [13], inducing cancer cell death [14], and treating oral cavity infections [15]. Additionally, several studies have explored the role of cold plasma in osteoblast differentiation [16]. Cold plasma treatment increases β-catenin expression after one day of differentiation in human periodontal ligament cells and elevates BMP2 and Runx2 levels after three days of differentiation. This process controls differentiation into osteoblasts through Wnt/β-catenin signaling, ultimately leading to increased levels of ALP and related factors, which are indicators of osteoblast differentiation [17].

Cold plasma emits various active species, such as reactive oxygen species (ROS) and hydroxyl radicals (∙OH), depending on the processing method, and ionizes gases using existing energy sources [18]. Cold plasma-generating gases include argon, helium, and oxygen, whose effects vary depending on the gas used [19]. Cold plasma processing methods are largely divided into direct and indirect methods, with different outcomes based on the processing method used. Direct treatment of microorganisms with cold plasma has a much faster effect than indirect treatment and has been shown to kill streptococci, staphylococci, and yeasts [20]. Additionally, studies on *Cutibacterium acnes* have shown that direct cold plasma treatment is more effective in killing bacteria than indirect plasma-activated water [20].

However, direct treatment methods result in the generation of higher ozone levels, reported at 57 ppm (compared to indirect methods) [12]. Although ozone has potential therapeutic applications, excessive amounts of it pose health risks. Moreover, ozone has been considered a toxic gas until the 16th century; however, it has recently been used for the potential treatment of various diseases [21]. Specifically, it is effective against skin diseases [22], oral diseases [23], and cancer [24]. Despite its potential, the safety of ozone remains a concern since acute exposure to it can cause serious problems such as decreased lung function and temporary abnormalities in respiratory function [25]. Accordingly, the Food and Drug Administration (FDA) has set the acceptable ozone generation standard for medical devices to <0.05 ppm over a specified period [26].

Therefore, in this study, we evaluated bone formation in mouse osteoblasts based on the plasma treatment method using the No-ozone Cold Plasma (NCP) device developed by our research team.

## 2. Materials and Methods

### 2.1. NCP Generation

**Figure 1:**
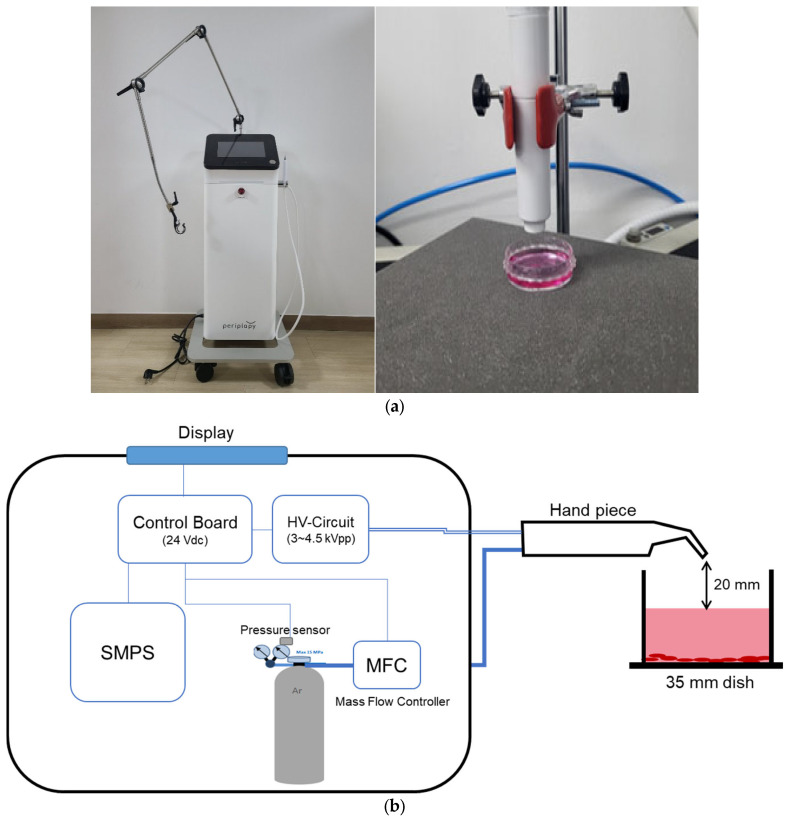
No-ozone Cold Plasma (NCP) device used in this study. (**a**) A photograph of the NCP device used to treat MC3T3-E1 cells. (**b**) Schematic of the NCP device. NCP, no-ozone cold plasma; SMPS, switched-mode power supply; HV, high voltage.

The NCP used in this experiment was generated using a device called Periplapy, developed by Feagle Co., Ltd. (Yangsan, Republic of Korea), which is a registered patent, trademark and design at the Korean Intellectual Property Office. The device was tested by the Korea Testing and Research Institute and generates a maximum ozone concentration of 0.008 ppm, nitrogen monoxide of <0.001 ppm, and nitrogen dioxide of 0.007 ppm. Figure 1 shows a photograph and a schematic diagram of the NCP device.

The device consists of a switched-mode power supply, a mainboard for control, a high-voltage circuit, a pressure sensor, and a regulator. It has an LCD panel and a handpiece as an attachment (hand-held). When the power button is pressed on, power is supplied to the main body, and argon gas is delivered to the handpiece at a constant flow rate. NCP is generated inside the nozzle of the handpiece when argon gas flows through it and AC high voltage is applied to the inner and outer electrodes of the nozzle. The NCP intensity can be set to one of three modes. In this study it was set to Mode 3 at 4.5 kVpp, 1.25 slm ± 20%. We opted for this experimental setting to maximize the effectiveness of the NCP treatment while keeping the duration brief.

### 2.2. Cell Culture

MC3T3-E1 cells were purchased from ATCC (Manassas, VA, USA). The MC3T3-E1 cells were cultured in α-MEM (Gibco BRL, Gaithersburg, MD, USA) containing 10% fetal bovine serum (FBS) (Gibco BRL), and 1% antibiotics (Gibco BRL) and incubated at 37 °C in an atmosphere of 5% CO_2_.

The following osteogenic differentiation induction medium was used for the osteoblast differentiation experiment: α-MEM supplemented with 10% FBS, 10 mM beta-glycerophosphate (Sigma-Aldrich, St Louis, MO, USA), 50 µg/mL ascorbic acid (Sigma-Aldrich), and 100 nM dexamethasone (Gibco BRL). Conventional media were used for testing the cell proliferation rates across all groups, while osteogenic induction media were used when differentiation began. The differentiation control group was treated with the osteogenic culture medium.

### 2.3. NCP Treatment

The cells were divided into the following four groups:

1. First group: not treated with NCP (NT);
2. Second group: treated directly with NCP in the dish with cells (DT);
3. Third group: treated directly with NCP in the dish, and the medium was immediately replaced (MC);
4. Fourth group: treated with NCP in a 35 mm dish without cells for 1 min, and then transferred to the dish with cells (PAM).

The treatment time for all NCP applications was 1 min, and the distance between the NCP device and the cells was 20 mm. All NCP groups were cultured after one treatment with NCP.

### 2.4. Water-Soluble Tetrazolium 1 (WST-1) Salt Assay

MC3T3-E1 cells were seeded at a density of 2 × 10^5^ cells in a 35 mm dish and incubated at 37 °C for 24 h. Each treatment group was treated with NCP and cultured for 24 or 48 h. Subsequently, the WST-1 solution (EX-Cytox, DoGenBio, Seoul, Republic of Korea) was diluted with media in a 1:9 ratio, and 1 mL of the resulting media was added to each well. After culturing in an incubator at 37 °C for 2 h, cell viability was measured using a microplate reader (Thermo Fisher Scientific, Darmstadt, Germany) at 450 nm [27]. All experiments were conducted independently and repeated in triplicate.

### 2.5. Alkaline Phosphatase (ALP) Staining

MC3T3-E1 cells were seeded at a density of 2 × 10^5^ cells in a 35 mm dish and incubated at 37 °C for 24 h. Each NCP-treated group was cultured in osteogenic medium for 1 or 2 weeks, with medium changes every 2–3 days. After incubation, the cells were fixed for 1 min using 3.7% formaldehyde (Sigma-Aldrich, St Louis, MO, USA) and 90% ethanol solution (Sigma-Aldrich, St Louis, MO, USA), followed by washing with TBS solution. Staining was performed in a dark room for 20 min with a fast 5-bromo-4-chloro-3-indolyl phosphate and nitroblue tetrazolium (BCIP/NBT) ALP substrate solution (B6404, Sigma-Aldrich, St Louis, MO, USA) [28]. Subsequently, the cells were washed four times with distilled water (DW) and photographed using a camera.

### 2.6. ALP Activity

MC3T3-E1 cells were seeded at a density of 2 × 10^5^ cells in a 35 mm dish and incubated at 37 °C for 24 h. Subsequently, each NCP-treated group was cultured in osteogenic medium for 2 weeks, with medium changes every 2–3 days. Cells were washed with phosphate buffered saline (PBS), sonicated in 0.1 M Tris buffer containing 0.5% Triton X-100 solution on ice at 4 °C, centrifuged at 14,000 rpm for 10 min. The supernatant was used for Bradford analysis. ALP was measured using a laboratory assay ALP kit (Wako Pure Chemicals, Osaka, Japan) and a microplate reader at 450 nm [29]. Data analysis was performed according to the manufacturer’s instructions.

### 2.7. Alizarin Red S Staining

MC3T3-E1 cells were seeded at a density of 2 × 10^5^ cells in a 35 mm dish and incubated at 37 °C for 24 h. Subsequently, each NCP-treated group was cultured in osteoinduction medium for 2 weeks, with medium changes every 2–3 days. After incubation, the cells were washed with PBS and fixed with 10% formaldehyde (Sigma-Aldrich) for 15 min. After washing twice with DW, the fixed cells were stained with 2% Alizarin Red S (pH 4.2) (Sigma-Aldrich) for 2 min, washed with DW, dried, and imaged using a camera. After imaging, the stained cells were exposed to 100 mM cetylpyridinium chloride (Sigma-Aldrich) for 2 h and the absorbance of the eluted supernatant was measured at 575 nm using a microplate reader [28].

### 2.8. Real-Time Polymerase Chain Reaction (qPCR)

MC3T3-E1 cells were seeded at a density of 2 × 10^5^ cells in a 35 mm dish and incubated at 37 °C for 24 h. Each NCP-treated group was cultured in osteoinduction medium for 2 weeks, with medium changes every 2–3 days. After incubation, total RNA was extracted using TRIzol reagent (Invitrogen, Carlsbad, CA, USA). cDNA was prepared by reverse transcription (One-step PreMix kit; iNtRON Biotechnology Inc., Seoul, Republic of Korea) on a T100 Thermal Cycler (Bio-Rad, Hercules, CA, USA), and PCR amplification was performed using a SensiFAT SYBR No ROX kit (Bioline, OH, USA) on a CFX 96 Real-Time System (Bio-Rad, Hercules, CA, USA). Relative quantification was achieved using the comparative 2^−ΔΔCt^ method. All samples were run in triplicate and normalized to the housekeeping gene, *GAPDH* [17]. The following primers were used: alkaline phosphatase-1 Forward, 5′-ACGAGGTCACGTCCATCCT-3′; alkaline phosphatase-1 Reverse, 5′-CCGAGTGGTGGTCACGAT-3′, osteocalcin Forward, 5′-ACAGACAAGTCCCACACAGCAACT-3′; osteocalcin Reverse 5′-CCTGCTTGGACATGAAGGCTTTGT-3′, GAPDH Forward 5′-TGGGAAGCTGGTCATCAAC-3′; GAPDH Reverse 5′-GCATCACCCCATTTGATGTT3-3′.

### 2.9. Statistical Analysis

All experiments were independently repeated three times (n = 3). The results were graphed using GraphPad Prism 5.0, and statistical analysis was performed using the SPSS (version 24, Chicago, IL, USA) statistical software package. Data analysis was performed using one-way ANOVA, followed by Duncan’s post hoc analysis. The different letters shown in the figures indicate significant differences among the groups after post-analysis. In other words, the difference between groups with the same letter is not statistically significant, whereas the difference between groups with different letters is statistically significant.

## 3. Results

### 3.1. Effect of NCP on MC3T3-E1 Cell Proliferation

**Figure 2:**
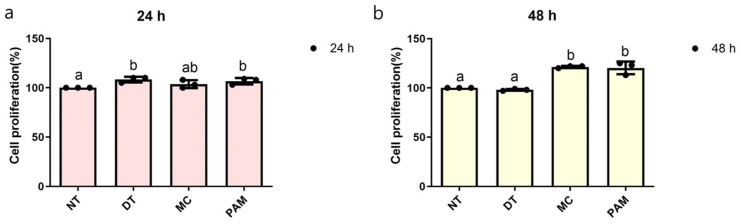
Cell proliferation of MC3T3-E1 cells treated with NCP through various methods. Cell proliferation rates after (**a**) 24 h and (**b**) 48 h of treatment. Different letters above the bars indicate significant differences between groups (n = 3 per each point in bar graph). NCP, no-ozone cold plasma; NT, control group that was not treated with NCP; DT, directly treated with NCP; MC, directly treated with NCP followed by medium replacement; PAM, indirectly treated with NCP-treated medium.

After 24 h of treatment with NCP, cells in the NT, DT, MC, and PAM groups exhibited proliferation rates of 100, 108, 103, and 106%, respectively (*p* < 0.038). After 48 h, the cell proliferation rates were 100, 98, 121, and 120% for the NT, DT, MC, and PAM groups, respectively (*p* < 0.000). At 24 h, the cell proliferation rates in the DT and PAM groups were significantly increased compared to the control (NT) group, whereas at 48 h, the MC and PAM groups showed a statistically significant increase compared to the NT group (Figure 2). Consequently, after 24 and 48 h, the PAM group showed the most significant effect on cell proliferation among all NCP methods, with none of them exhibiting cytotoxicity.

### 3.2. ALP Activity in the NCP-Treated MC3T3-E1 Cells According to NCP Treatment Method

**Figure 3:**
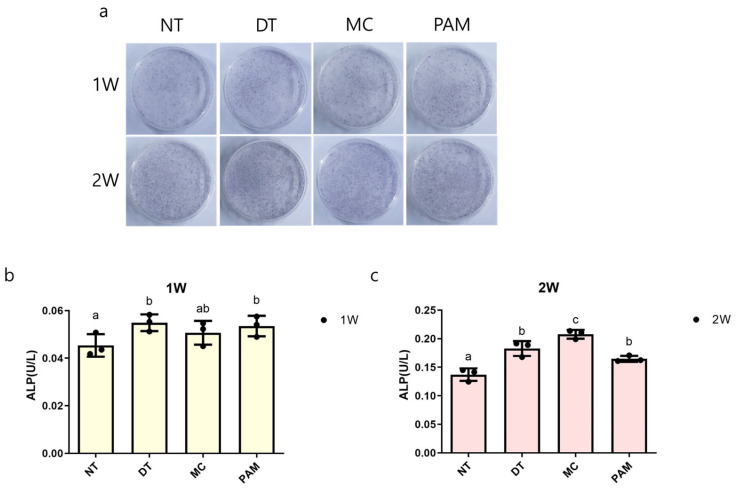
ALP activity in osteoblasts after NCP treatment. (**a**) ALP staining images of MC3T3-E1 cells and (**b**) measurements of ALP activity at 1 week and (**c**) 2 weeks of NCP treatment using various methods. Different letters above the bars indicate significant differences between groups (n = 3 per each point in bar graph). NCP, no-ozone cold plasma; ALP, alkaline phosphatase; NT, control group that was not treated with NCP; DT, directly treated with NCP; MC, directly treated with NCP followed by medium replacement; PAM, indirectly treated with NCP-treated medium.

ALP staining was increased in the DT, MC and PAM groups compared to the NT group at 1 week. The MC group exhibited increased staining even at 2 weeks. Moreover, an increase in ALP activity was observed in the DT and PAM groups compared to the untreated group at 1 week (*p* < 0.115). The DT, MC, and PAM groups showed a statistically significant increase at 2 weeks (*p* < 0.000) compared to the NT group, with the highest activity observed in the MC group (Figure 3).

### 3.3. Calcium Deposition in NCP-Treated MC3T3-E1 Cells

**Figure 4:**
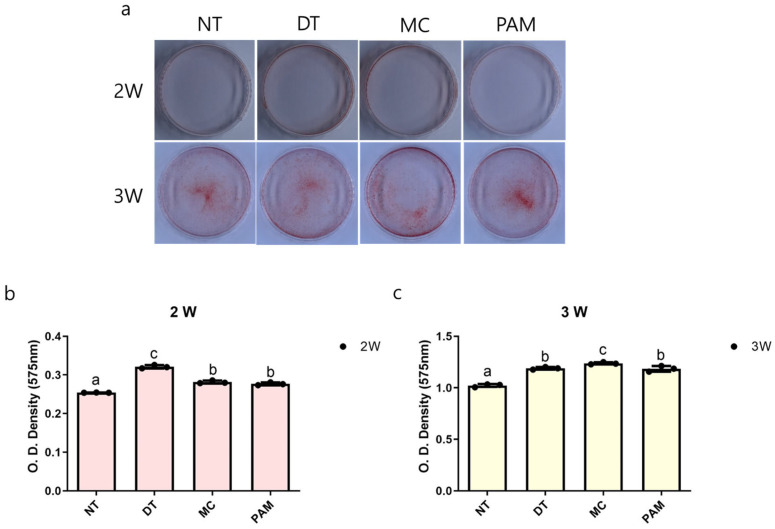
Alizarin Red S staining results of osteoblasts treated with NCP. (**a**) Photographs of MC3T3-E1 cells after Alizarin Red S staining and (**b**,**c**) optical density measurements of Alizarin Red S of cells treated with NCP at 2 and 3 weeks using various methods. Different letters above the bars indicate significant differences between groups (n = 3 per each point in bar graph). NCP, no-ozone cold plasma; OD, optical density; NT, control group that was not treated with NCP; DT, directly treated with NCP; MC, directly treated with NCP followed by medium replacement; PAM, indirectly treated with NCP-treated medium.

Alizarin Red S staining did not show a significant visual difference between the groups at 2 and 3 weeks. However, OD values of eluted stained cells indicated increased calcium deposition in the DT, MC, and PAM groups at 2 and 3 weeks compared to the NT group (*p* < 0.000). The highest deposition was observed in the DT group at 2 weeks and the MC group at 3 weeks (Figure 4).

### 3.4. mRNA Expression Changes in NCP-Treated MC3T3-E1 Cells

Real-time PCR analysis showed that ALP mRNA expression levels were lower in the DT and MC groups than in the NT and PAM groups at 1 week (*p* < 0.000). Additionally, the MC group had the highest levels of ALP mRNA expression compared to the other groups at 2 weeks (*p* < 0.014). No significant difference was observed between the groups at 3 weeks (*p* < 0.293).

**Figure 5:**
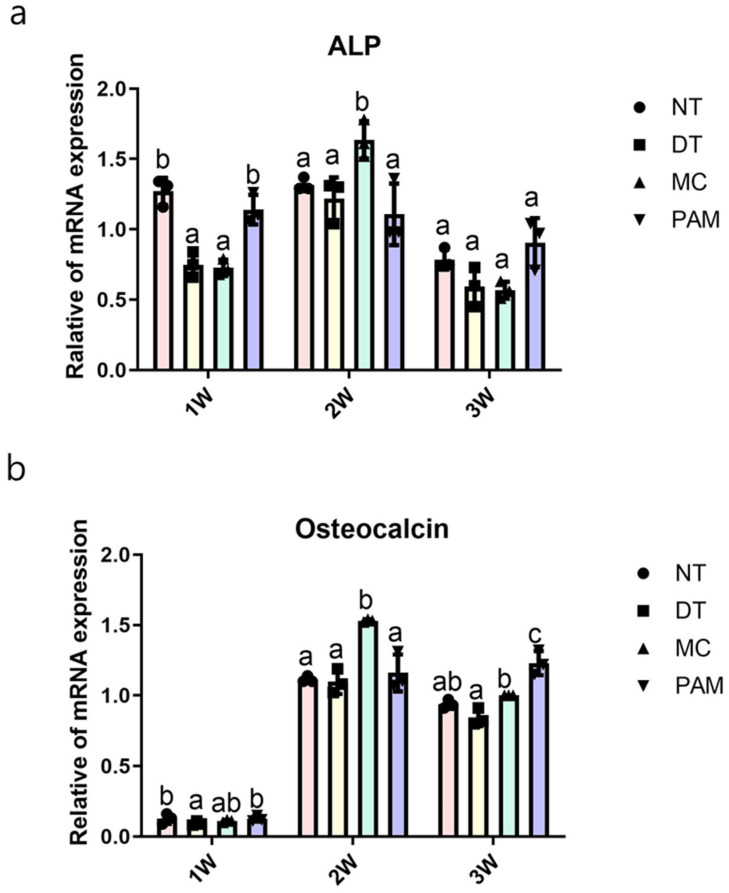
Real-time PCR analysis results of (**a**) ALP and (**b**) osteocalcin expression in MC3T3-E1 cells treated with NCP using various methods. Different letters above the bars indicate significant differences between groups (n = 3 per each point in bar graph). NCP, no-ozone cold plasma; ALP, alkaline phosphatase; NT, control group that was not treated with NCP; DT, directly treated with NCP; MC, directly treated with NCP followed by medium replacement; PAM, indirectly treated with NCP-treated medium.

The DT group showed lower osteocalcin expression compared to the NT and PAM groups at 1 week (*p* < 0.109); however, no significant difference was observed compared to the MC group. The MC group showed the highest osteocalcin expression compared to the other groups at 2 weeks (*p* < 0.000), similar to the ALP results. In contrast, the PAM group showed the highest osteocalcin expression at 3 weeks, followed by the MC group (*p* < 0.000). Although the MC and DT groups showed significant differences, neither showed significant differences compared to NT in the post-test. Statistically significant differences in ALP expression were observed at weeks 1 and 2, and in osteocalcin expression at weeks 2 and 3 (Figure 5). Our results showed that the expression of ALP and osteocalcin increased in all NCP treatment groups except the DT group. This suggests that, among the NCP treatment groups, MC and PAM treatment methods promoted differentiation into osteoblasts more effectively.

## 4. Discussion

Bone remodeling occurs continuously throughout life, involving osteoblasts that form bone osteoclasts that resorb bone [30]. However, osteoporosis occurs when the balance between bone formation and resorption is disrupted due to aging or disease, leading to weakened bones. Therefore, in this study, we compared the effects of direct and indirect cold plasma treatments on osteoblast stimulation, focusing on cell proliferation and differentiation.

When cells are treated with cold plasma, the effects differ depending on the treatment method (direct or indirect). Direct NCP treatment involves charged particles, such as argon ions and electrons, directly impacting cells. The charged particles accumulate on the outer surface of the cell membrane. For instance, they destroy the cell membrane and cell wall when exposed to bacteria, leading to bacterial inactivation [31].

However, during indirect treatment, NCP is applied directly to liquid-like media, which generates chemical elements such as ROS and reactive nitrogen species (RNS) within the media. The cells are then indirectly treated with NCP by exposing them to the media, which may lead to cell damage. The various active species, including ROS and RNS, are effective against cancer cells [32]. Although low levels of ROS are essential for cell proliferation and growth, excessive accumulation of ROS within healthy cells ultimately leads to cell death [33]. Therefore, an appropriate cold plasma treatment method for inducing cell activity without causing cell damage is crucial.

Previous studies have shown that NCP can regulate E-cadherin in skin cells, indicating the significant role of charged particles in plasma [34]. However, OH and NO do not have significant effects [34]. Similarly, a study on oral bacteria highlighted the importance of charged particles in bacterial inactivation [35].

In this study, cells were treated with direct and indirect plasma treatment. In particular, the cell medium was exchanged as a method to minimize the influence of the indirect medium treated with plasma among the direct treatment methods (MC). We evaluated the effects of NCP on osteoblasts using different treatment methods and found that the cell proliferation rate increased in all experimental groups during the 24 h cell culture period without cytotoxicity. After 48 h, the MC and PAM groups showed a statistically significant increase (about 20%) in proliferation rate compared to the NT group. These findings align with the results of a previous study in which human periodontal ligament cells were treated with NCP [17].

ALP is an indicator of the early stage of osteoblast differentiation [36]. It is secreted into the extracellular matrix and can be observed through cellular staining or mRNA levels. As bone begins to form, the activity of extracellular matrix proteins such as osteocalcin, an indicator of mature osteoblasts, increases, and mineral crystals such as calcium are deposited. These calcium deposits can be observed through Alizarin Red S staining [37]. In this study, the NCP-treated mouse osteoblasts in the MC group showed stronger positive cytochemical labeling for ALP and higher ALP activity than those in the other groups. This is consistent with other studies that showed that ALP levels usually increase to a maximum at week 2 [38].

Calcium deposition experiments using ARS staining yielded similar results, with a significant increase in the number of mineralized nodules in the MC group at 3 weeks. These findings suggest that direct NCP treatment enhances osteoblast differentiation owing to increased ALP activity. However, ALP mRNA levels in the MC group were initially lower than in the NT group at week 1 but increased at week 2, indicating a peak in ALP levels at 2 weeks. Therefore, our results suggest that direct NCP treatment, followed by media replacement, is the optimal treatment method that leads to increased ALP levels. Similarly, osteocalcin levels increased in the MC group at 2 weeks, and PAM levels were the highest at 3 weeks. In another study, osteocalcin exhibited as the differentiation period progressed until the fourth week [39]. Conversely, we only analyzed the results up to the third week. Therefore, a longer analysis period will be required in the future.

In summary, the findings from this study demonstrated that NCP treatment, both direct and indirect, induces mouse osteoblast differentiation. The direct treatment in the MC group was the most effective method. These findings hold promise for treating patients with osteoporosis or bone defects. Additionally, because the production of ozone is minimized, it can be safely used in the human body when developed as a medical device.

The limitations of this study include the small sample size and the lack of a mechanism to explain the osteoblast differentiation effect observed in each NCP group. Additionally, as this study was conducted in vitro, its applicability to humans is limited. Therefore, further in vitro and in vivo studies are necessary to elucidate the process of controlling bone formation using NCP.

## 5. Conclusions

Direct and indirect NCP treatments of mouse-derived osteoblasts increase cell proliferation and promote osteoblast differentiation. Direct (DT, MC) and indirect (PAM) treatment effects varied, with MC treatment having a more significant impact on osteoblast activity. Enhanced cell differentiation into osteoblasts was observed in the MC group, indicating that direct NCP treatment followed by media replacement was the most effective treatment method to improve bone formation in vitro.

## 6. Patents

The Periplapy (Feagle Co., Ltd., Yangsan, Republic of Korea) is registered as a patent (10-2568541), design (30-1171975-0000) and trademark (401-824232-0000) at the Korean Intellectual Property Office.

## Data Availability

The data presented in this study are available upon request from the corresponding author.
